# Remarks on the Maximum Entropy Principle with Application to the Maximum Entropy Theory of Ecology

**DOI:** 10.3390/e20010011

**Published:** 2017-12-27

**Authors:** Marco Favretti

**Affiliations:** Dipartimento di Matematica “Tullio Levi-Civita”, Università degli Studi di Padova, 35122 Padova, Italy; favretti@math.unipd.it; Tel.: +39-049-827-1408

**Keywords:** Maximum Entropy principle, Maximum Entropy Theory of Ecology, Shannon entropy, Boltzmann counting

## Abstract

In the first part of the paper we work out the consequences of the fact that Jaynes’ Maximum Entropy Principle, when translated in mathematical terms, is a *constrained extremum problem* for an entropy function H(p) expressing the uncertainty associated with the probability distribution *p*. Consequently, if two observers use different independent variables *p* or g(p), the associated entropy functions have to be defined accordingly and they are different in the general case. In the second part we apply our findings to an analysis of the foundations of the Maximum Entropy Theory of Ecology (M.E.T.E.) a purely statistical model of an ecological community. Since the theory has received considerable attention by the scientific community, we hope to give a useful contribution to the same community by showing that the procedure of application of MEP, in the light of the theory developed in the first part, suffers from some incongruences. We exhibit an alternative formulation which is free from these limitations and that gives different results.

## 1. Introduction

Maximum Entropy Principle MEP (E.T. Jaynes, 1957, see [1,2]) is a powerful inference principle which allows to determine the probability distribution that describes a system on the basis of the information available, usually in the form of averages of observables (random variables) of interest for the system. It is based on: (i) the enumeration of the system states i=1,…,N; (ii) the introduction of one or more functions that translate the information available of the system in the form of constraints on the probability i.e., as f(p)=c where *c* is a vector of average values; and (iii) a function measuring the *uncertainty* associated to a probability distribution candidate to describe the system. We consider here only systems with a finite number of states since the extra generality of considering an infinite number of states is not necessary for the aims of this work. The sought distribution is the one for which uncertainty is maximal on the set of distributions for which f(p)=c. This distribution thus represents the least biased estimate on the basis of the given information whether or not its prescriptions are empirically confirmed by experiments. Each the above outlined Steps (i)–(iii) has a profound meaning and impact on the implementation of the MEP procedure. For example, if the states of the system are a priori equally probable, the uncertainty function to use is Shannon entropy H(p), while if they are statistically described by a prior distribution *q* the uncertainty function is (minus) the relative entropy D(p|q). If the systems states are the result of a coarse-graining procedure from a bigger set of a priori equiprobable states, then a prior distribution *q* which counts the number of coarse-grained states has to be used for D(p|q) in order to render the MEP procedure invariant with respect to the coarse graining as explained in [3]. See also [4] where a number of different probability distribution used in ecology are derived for a system of individuals arranged in different cells by simply making different assumptions on the individuals (indistinguishable or not), on the constraints and on the coarse graining. The variety of MEP responses simply reflects the facts that different pieces of information are considered; if the previsions fail to be confirmed by the experiment, this is the signal that relevant information for the statistical description on the system under study has been neglected.

A basic tenet of the Maximum Entropy Principle is that two observer which are given the same information expressed by Steps (i) and (ii) above must obtain the same result upon application of the MEP, i.e., the solution to the constrained extremum problem has to be unique. Notice that the MEP procedure does not specify the probability $p=(p_{1},\ldots ,p_{N})$ to use; thus if one observer uses *p* and another uses $p^{\prime }$ which is related to *p* by a one-to-one transformation $p=g(p^{\prime })$, provided that they set up the constraints in the form f(p)=c and $f(g\left(p^{\prime }\right))=c$ respectively, they are translating in mathematical terms the same information on the system. This represents the same degree of freedom of using different (but related by an invertible transformation) systems of coordinates to describe the position of a point in physics. Therefore, to ensure that the results of the application of the MEP procedure are independent of the choice of the *p* used the uncertainty function has to be defined accordingly. Proposition 1 in Section 2 below is the main tool to determine the form of the uncertainty function using *g*-related distributions.

The analysis of this subtile point of the application of MEP is the main aim of this paper. We argue that since there seems not to be a preferred choice for *p* they have to be considered all equivalent; therefore the related uncertainty functions are also equivalent and can be derived by a single given one. The question is: which is the translation in mathematical terms of the initial information which is entitled to use as uncertainty function the Shannon entropy? We give an answer by resorting to the Boltzmann statistical derivation of the entropy function, which has the same form of Shannon one. This statistical procedure, based on independent tosses of indistinguishable particles in bins, can be applied to different models and allows determining from a sort of “first principle” the form of the entropy function to use. We think that there is room for further investigation of this subtile issue of the application of MEP.

In the second part of the paper we apply the above findings to a specific application of MEP to ecology called Maximum Entropy Theory of Ecology (M.E.T.E.) as exposed in [5]. M.E.T.E. is a purely statistical model of an ecological community. Using the Maximum Entropy Principle the theory aims at inferring the form of some of the most used distributions in ecology from the knowledge of macroscopic information on the system: number of individuals, number of species and total metabolic requirement. MEP has been applied in the field of statistical ecology mainly to derive one of the most important probability distributions, the Species Abundance Distribution (SAD) but not exclusively (see [6] or [7] where it is used for modeling species geographic distributions with presence-only data). Several papers ([3,8,9]) have revised the application of the MEP inference principle to ecological problems, stressing the importance of the choice of the system’ coordinate [10,11,12], of the prior distribution [4,8] and of the entropy function [3,6]. The interest of [5] is that it considers simultaneously information on the distribution of individuals among species (SAD) and on the distribution of metabolic energy rate among individuals. In this richer scenario a number of probability distributions for different observables of interest are derived. The output of the work has grown to become a comprehensive theory of application of MEP to ecological problems, called Maximum Entropy Theory of Ecology (see the book [13,14]) which has been extensively tested with real ecosystem data in [15,16,17]. In Section 4.1 we review the assumptions of the model in [5] and the related MEP solution; in Section 4.2 we propose an alternative application of the MEP procedure starting from the same initial information but giving a different result. In Section 5 we discuss the non equivalence of the two procedures using the Proposition 1 below and motivate why the MEP solution in [5] is flawed by some inconsistences.

## 2. MEP Formulation Using Different Variables

The Maximum Entropy Principle is generally expressed as: *p* is the distribution that maximizes Shannon entropy H(p) over the set of distributions allowed by the constraints. Let $p^{\prime }$ defined by $p^{\prime }=g^{-1}\left(p\right)$ with *g* invertible be a different choice of the variable to be used to express the probability distribution and the constraints and suppose that for the solution of this constrained extremum problem we use Lagrange multipliers method. Then, the following Proposition holds (see Appendix A for a proof).

**Proposition** **1.** $\hat{p}$ is the constrained extremum of H(p) over the set f(p)=c if and only if ${\hat{p}}^{\prime }=g^{-1}\left(\hat{p}\right)$ is the constrained extremum of $H(g\left(p^{\prime }\right))$ over the set $f(g\left(p^{\prime }\right))=c$.

From the above Proposition 1 we learn that if we are given the entropy function and the constraints as (p,f(p),H(p)) and we want to use another variable $p^{\prime }=g^{-1}\left(p\right)$, to find the same MEP solution we have to transform the constraints *and* the entropy function accordingly using $\left(p^{\prime },f\circ g\left(p^{\prime }\right),H\circ g\left(p^{\prime }\right)\right)$. In this way, changing variable and finding the maximum are operations whose order can be interchanged. A natural question arises in this setting: which one is the couple (p,f(p)) translating in mathematical terms the initial information on the system that “is justified” in using the entropy function in Shannon form? We will give a partial answer to this question in Section 3 below.

Note that the invariance requirement with respect to the choice of the independent variable is different from the requirement, introduced in the axiomatic derivation of MEP in [18], of invariance *in the form* of H(p) with respect to a transformation of the type y=Γ(x), where x∈D are the (possibly infinite) states of a system and p=p(x). For a system with finite states the transformation Γ (called coordinate transformation in [18]) reduces to a relabeling of the states. Shannon entropy $H\left(p\right)=-\sum_{i=1}^{N}p_{i}\ln p_{i}$ being additive is clearly invariant in form, expressing the fact that different observers may use different labels for the *N* system states. This relabeling transformation

$$p_{k}^{\prime }=g_{k}\left(p_{1},\ldots ,p_{N}\right)=p_{\varphi_{k}\left(1,\ldots ,N\right)}\;k=1,\ldots ,N$$

where $\varphi_{k}$ is a permutation of the *N* state labels is thus a particular case of the more general *g* considered in the above Proposition 1. It is clearly an invertible transformation in which H(p) and $H(g\left(p^{\prime }\right))$ do coincide, but this is no longer assured for more general transformations *g*; see Section 3 below.

## 3. A Simple, Ecologically Oriented Example

To throw light on the questions considered above we take a very simple system used in a number of physical models that for our purposes can be considered as a minimal example of an ecological community. Consider a set I of $N_{0}$ identical individuals (balls) which belong to *S* species (indistinguishable urns). Suppose that each species contains at least one individual so that the maximal number of individuals allowed to one species is $N=N_{0}-\left(S-1\right)$. Suppose that the species labels can be interchanged so that the observable quantity is not the number of individuals $n_{\alpha }$ of a given species α but the number $S_{n}$ of species which have exactly *n* individuals, for n=1,…,N.

Suppose that the set of species S is equipped with the uniform probability 1/S and consider the random variable ν:S→N, $\alpha \mapsto \nu \left(\alpha \right)=n_{\alpha }$, N={1,…N} that assigns to a species its abundance. Let ϕ(n) be the density associated to the random variable ν (in the sequel *P* denotes probability)

(1) $$\phi \left(n\right)=P\left(\nu =n\right)=P\left(\alpha \in \nu^{-1}\left(n\right)\right)=\frac{|\nu^{-1}\left(n\right)|}{S}=\frac{S_{n}}{S},\;n\in N$$

where |A| is the number of elements of *A*. Set for later use $S_{n}=\nu^{-1}\left(n\right)\subset S$, therefore $|S_{n}|=S_{n}$.

Let the set of individuals I be equipped with the uniform probability $1/N_{0}$, let α:I→S, i↦α(i) be the assignation of each individual to a species and consider the random variable ν∘α:I→N, i↦ν(α(i)). Moreover, consider the following subset $I_{n}$ of I obtained by grouping together all the individuals belonging to the species having *n* individuals (we call this subset the “multispecies” *n*)

(2) $$I_{n}=\left\{i\in I:\nu \left(\alpha \left(i\right)\right)=n\right\}\subset I,\;I_{n}=\left|I_{n}\right|.$$

As a straightforward consequence, we have that $I_{n}=nS_{n}$. Let $\phi_{I}\left(n\right)$ be the density associated to the random variable ν∘α that is

(3) $$\phi_{I}\left(n\right)=P\left(\nu \circ \alpha =n\right)=P\left(i\in I_{n}\right)=\frac{I_{n}}{N_{0}},\;n\in N.$$

Both ϕ and $\phi_{I}$ are probability distributions on N; it is easy to see that since $I_{n}=nS_{n}$, they are related by an invertible transformation $\phi_{I}=g\left(\phi \right)$ defined as

(4) $$\phi_{I}\left(n\right)=g_{n}\left(\phi \right)=\frac{nS}{N_{0}}\phi \left(n\right)\;n=1,\ldots ,N.$$

In ecology the distribution ϕ is called Species Abundance Distribution (SAD) and is probably the most important (and investigated) indicator of how an ecological community is structured. While ϕ(n) gives the probability of extracting a species of abundance *n* from the set of species, $\phi_{I}\left(n\right)$ gives the probability that an individual extracted from the set I belongs to a species of abundance *n*. Real communities are composed of many species with few individuals (rare species) and few ones which contain the majority of the individuals (common species). As a consequence, the two probability distribution are very different.

The information on the system resumed by the numbers $N_{0},S$ sets the following equalities

(5) $$N_{0}=\sum_{n=1}^{N}nS_{n}=\sum_{n=1}^{N}I_{n}\;\mathrm{and}\;S=\sum_{n=1}^{N}S_{n}=\sum_{n=1}^{N}\frac{1}{n}I_{n}.$$

If we divide the above two equalities by *S* or $N_{0}$ respectively, we obtain the following equations to be satisfied by ϕ

(6) $$\frac{N_{0}}{S}=\sum_{n=1}^{N}n\phi \left(n\right),\;1=\sum_{n=1}^{N}\phi \left(n\right)$$

and $\phi_{I}$ respectively

(7) $$1=\sum_{n=1}^{N}\phi_{I}\left(n\right),\;\frac{S}{N_{0}}=\sum_{n=1}^{N}\frac{1}{n}\phi_{I}\left(n\right).$$

Constraints (6) and (7) are *g*-related, that is ϕ satisfies Equation (6) if and only if g(ϕ) satisfies Equation (7). Note that in first case we we are constraining the weighted *arithmetic* average of 1,…,N while in second one we are constraining the weighted *harmonic* average. Note also that the actual information being used is the *ratio* $N_{0}/S$ or equivalently $S/N_{0}$, so that the MEP description of the system is independent of the size $N_{0},S$ of the system provided that the ratio $N_{0}/S$ is kept constant.

Suppose that two different observers translate in mathematical terms the information contained in the data $N_{0},S$ using the two above introduced variables ϕ and $\phi_{I}$ and that both invoke the MEP to find the least informative probability distribution allowed by the constraints. The application of MEP with entropy function $H\left(\phi \right)=-\sum_{n=1}^{N}\phi \left(n\right)\ln\phi \left(n\right)$ and Constraint (6) gives, upon application of the Lagrange multipliers methods, the geometric probability distribution

(8) $$\hat{\phi }\left(n\right)=\frac{e^{-\lambda n}}{Z(\lambda )},\;Z\left(\lambda \right)=\sum_{n=1}^{N}e^{-\lambda n}$$

while the MEP procedure with *the same* entropy function $H\left(\phi_{I}\right)=-\sum_{n=1}^{N}\phi_{I}\left(n\right)\ln\phi_{I}\left(n\right)$ and Constraint (7) gives the solution

(9) $${\hat{\phi }}_{I}\left(n\right)=\frac{e^{-\gamma \frac{1}{n}}}{Z(\gamma )},\;Z\left(\gamma \right)=\sum_{n=1}^{N}e^{-\gamma \frac{1}{n}}$$

whose corresponding SAD is

(10) $$\tilde{\phi }\left(n\right)=g^{-1}\left({\hat{\phi }}_{I}\right)\left(n\right)=\frac{N_{0}}{Sn}\frac{e^{-\gamma \frac{1}{n}}}{Z(\gamma )}.$$

It is easy to see that $\hat{\phi }\neq g^{-1}\left({\hat{\phi }}_{I}\right)$, therefore with this example we have shown that changing independent variables and using the MEP with the Shannon entropy are *non commuting operations* in the general case. Had we used Constraint (7) with the transformed entropy

(11) $$H^{\prime }\left(\phi_{I}\right)=H\left(g^{-1}\left(\phi_{I}\right)\right)=-\sum_{n=1}^{N}\phi_{I}\left(n\right)\frac{N_{0}}{nS}\ln\left[\phi_{I}\left(n\right)\frac{N_{0}}{nS}\right]$$

we would have obtained the solution $g(\hat{\phi })$ with $\hat{\phi }$ in Equation (8). Note that the transformed entropy in Equation (11) does not have the form of a relative entropy function therefore it can not be obtained by introducing a suitable prior probability distribution. Note also that the non commutativity considered above is different from the non commutativity of MEP procedure with respect to *coarse graining* of the system state considered in [3,4].

### Which One is the Correct Application of MEP?

Both ϕ(n) in Equation (8) and $\tilde{\phi }\left(n\right)$ in Equation (10) result from the application of the MEP starting from the same initial information $N_{0},S$ on the system and provide non equivalent answers to the question of how the abundance of individuals is distributed among the species. Since the set of distributions allowed by the constraints (feasible set) in the two formulations Equations (6) and (7) are related by an invertible transformation $D_{I}=g\left(D\right)$, the non equivalence must reside in the choice of the form of the entropy functions which are not *g*-related according to Proposition 1. On the basis of the same Proposition, once the form of the entropy function is established in a given formulation of MEP (p,f(p),H(p)), it is also determined for all *g*-related formulations. It seems thus necessary to invoke a sort of first principle to establish the correct form of the entropy function in the initial MEP formulation.

A possible approach could be to compare the form of the resulting probability distribution against field data. This is not entirely satisfactory since the discrepancy between the MEP solution and the empirical curve could be due to the fact that we have neglected (or we are not aware of) some information on the system which is relevant for the question addressed. In this case the discrepancy between the model and the empirical pattern is a signal that other factors shaping the probability distribution are acting on the system. By the way, for the problem considered above, it is generally acknowledged in the ecological literature (see e.g., [5]) that the geometric SAD in Equation (8) has a poor fit with the empirical SAD, while the one introduced in (10) could provide a better fit since is more flexible (it can be monotonically decreasing or display an interior mode depending on the value of the Lagrange multiplier).

At least for systems with a discrete number of states another criterion can be introduced, which uses the combinatorial argument providing one of the justifications for the form of Shannon entropy historically preceding the axiomatic derivation in [18].

This is the celebrated Boltzmann problem of statistical mechanics [19]. It is useful to briefly review its solution here because it tells us how to determine the correct form of the entropy function from first principles in cases where its determination is not obvious. It deals with the distribution of *K* indistinguishable particles (individuals) among *M* equally spaced energy levels ϵ=1,…,M. Boltzmann original formulation dealt with arbitrarily spaced energy levels but this extra generality is not needed here. If $n_{\epsilon }$ is the number of particles having energy ϵ and $n=(n_{1},\ldots ,n_{M})$ is the vector of occupation numbers of the energy levels, the logarithm of the probability of n is

(12) $$\ln W\left(n\right)=\ln\frac{1}{M^{K}}\frac{K!}{n_{1}!\ldots n_{M}!}$$

which using Stirling approximation lnn!≈nlnn can be rewritten as

(13) $$\ln W\left(n\right)=K\ln K-\sum_{\epsilon }n_{\epsilon }\ln n_{\epsilon }.$$

The maximization of lnW(n) under the constraints $\sum_{\epsilon }n_{\epsilon }=K$ and $\sum_{\epsilon }\epsilon n_{\epsilon }=E_{0}$ gives the most common, i.e., least informative vector of occupation numbers. The solution of the constrained maximization problem is the celebrated Boltzmann-Gibbs distribution

(14) $$n_{\epsilon }=\frac{Ke^{-\beta \epsilon }}{\sum_{\epsilon =1}^{M}e^{-\beta \epsilon }},\;\psi \left(\epsilon \right)=\frac{n_{\epsilon }}{K}=\frac{e^{-\beta \epsilon }}{\sum_{\epsilon =1}^{M}e^{-\beta \epsilon }}$$

Note that writing $n_{\epsilon }=\psi \left(\epsilon \right)K$, lnW in Equation (13) coincides up to a constant with

(15) $$H\left(\psi \right)=-\sum_{\epsilon }\psi \left(\epsilon \right)\ln\psi \left(\epsilon \right).$$

In the same manner, if we have *S* indistinguishable species to be assigned in the *N* levels (abundances) of N let $s=(S_{1},\ldots ,S_{N})$ be the occupation vector with constraints $\sum_{n}S_{n}=S$ and $\sum_{n}nS_{n}=N_{0}$. As before, setting $S\phi \left(n\right)=S_{n}$ the least informative probability distribution is

(16) $$\phi \left(n\right)=\frac{e^{-\lambda n}}{\sum_{n}e^{-\lambda n}}=\frac{e^{-\lambda n}}{Z(\lambda )}$$

and the associated entropy is

(17) $$H\left(\phi \right)=-\sum_{n}\phi \left(n\right)\ln\phi \left(n\right)$$

Therefore the form of the entropy function for the formulation given by Equation (6) of the MEP problem using the variable ϕ can be derived by Boltzmann combinatorial argument.

Let us see which is the resulting entropy function if we apply Boltzmann argument to the formulation given by Equation (7) using $\phi_{I}$. Remember that

(18) $$\left(A\right)\;\phi \left(n\right)=Prob\left(\alpha \in S_{n}\right),\;\;\;\left(B\right)\;\phi_{I}\left(n\right)=Prob\left(i\in I_{n}\right)$$

so basically in (A) we are “tossing” *S* species in the *N* bins $S_{n}$ while in (B) we are “tossing” $N_{0}$ individuals in the *N* multispecies $I_{n}$. In the Boltzmann argument the *tosses are supposed to be independent* and this is true for (A) but not for (B). Indeed a multispecies $I_{n}$ is the grouping of $S_{n}$ species each having *n* individuals so the number of individuals in $I_{n}$ can be updated only by multiples of *n*, i.e., adding or subtracting a species of *n* individuals. Hence the tosses of single individuals does not represent independent tosses. If we want to support the choice of the entropy function by Boltzmann argument we have to consider *tosses of groups* of *n* individuals. Let $i_{n}$ be the number of individuals tossed in $I_{n}$. Therefore the related occupation vector has the form

(19) $$i=\left(\frac{i_{1}}{1},\ldots \frac{i_{n}}{n},\ldots \frac{i_{N}}{N}\right),\;W\left(i\right)=\frac{1}{N^{N_{0}}}\frac{N_{0}!}{\frac{i_{1}}{1}!\ldots \frac{i_{N}}{N}!}$$

which leads to an entropy (recall that $i_{n}=N_{0}\phi_{I}\left(n\right)$) of the form

$$H\left(\phi_{I}\right)=-\sum_{n}\frac{N_{0}}{n}\phi_{I}\left(n\right)\ln\left[\frac{N_{0}}{n}\phi_{I}\left(n\right)\right]$$

and under the Constraints (7) above to the solution

(20) $$\phi_{I}\left(n\right)=n\frac{S}{N_{0}}\frac{e^{-\lambda n}}{Z(\lambda )}=g\left(\hat{\phi }\left(n\right)\right).$$

We have shown that in the case where the probability distribution being used can be linked at least conceptually to independent repetitions of an experiment, we have a criterion to derive the correct form of the entropy function from a first principle. Of course to build a general theory this simple example deserves further generalization.

## 4. Maximum Entropy Theory of Ecology

### 4.1. Review of M.E.T.E. Assumptions

Let us review the assumptions of M.E.T.E. as exposed in [5]. In [5], various metrics (probability distributions) are derived but here we will content ourselves with the analysis of the derivation of two of them, namely the Species Abundance Distribution ϕ(n) and the distribution of metabolic energy ψ(ϵ) between the individuals.

Following [5], we consider a community made of $N_{0}$ individuals living on an area $A_{0}$, subdivided into *S* species and having total metabolic energy rate $E_{0}$. Assume that each species has at least one individual and that the individual metabolic rate is a discrete quantity which ranges from 1 to a maximum *M* in a suitable energy unit. With these assumptions, the abundance of a species α is ν(α)=1,…N with $N=N_{0}-S+1$ and the individual metabolic rate is m(i)∈M={1,…,M}, with $M=E_{0}-S_{0}+1$. Each individual is labelled by a double label (α,ϵ) indicating the species α=1,…,S and the metabolic rate ϵ=1,…,M. Here we depart from [5] by assuming for the sake of simplicity that the individual metabolic rate is a discrete quantity while in [5] it is assumed to be a continuous one. This change does not affects our argument since for dealing with the continuous case it is enough to substitute the sums with integrals.

The information on the system is limited to the values $N_{0}$, *S*, $E_{0}$, and $A_{0}$, but for our aims the knowledge of $A_{0}$ is not relevant. Therefore we are dealing with a non spatial model of a community of *S* non interacting species. On the basis of this sole knowledge we would like to answer to two questions: ($Q_{1}$) How is the energy distributed among the individuals? ($Q_{2}$) How are the individuals distributed among the species? Note that, since the species’ label can be exchanged, the correct formulation of ($Q_{2}$) is how many of the species have exactly *n* individuals for n=1,…,N, which is the information contained in ϕ(n).

Both questions can be answered in a non deterministic manner; for ($Q_{2})$, we use the density ϕ introduced in Equation (1), while, for ($Q_{1})$, we introduce the map m:I→M, i↦m(i) that we will consider as a random variable and its density ψ(ϵ) on M

(21) $$\psi \left(\epsilon \right)=P\left(m=\epsilon \right)=P\left(i\in m^{-1}\left(\epsilon \right)\right)=\frac{I_{\epsilon }}{N_{0}},\;\forall \epsilon \in M$$

where $I_{\epsilon }=m^{-1}\left(\epsilon \right)\subset I$ and $I_{\epsilon }=\left|I_{\epsilon }\right|$. For later use, consider also the joint probability distribution

(22) $$P\left(n,\epsilon \right)=Prob\left(i\in I_{n},i\in I_{\epsilon }\right)=\frac{1}{N_{0}}\left|I_{n}\cap I_{\epsilon }\right|$$

having marginals, respectively, $\phi_{I}\left(n\right)$ and ψ(ϵ), since the space I is doubly partitioned in multispecies and energy classes.

### 4.2. Our Solution of M.E.T.E. Problem

The following constraints contain all the information $\left(N_{0},S,E_{0}\right)$ available on the system

(23) $$\sum_{\epsilon }\psi \left(\epsilon \right)=1,\;\sum_{\epsilon }\epsilon \psi \left(\epsilon \right)=\frac{E_{0}}{N_{0}}$$

(24) $$\sum_{n}\phi \left(n\right)=1,\;\sum_{n}n\phi \left(n\right)=\frac{N_{0}}{S}$$

and a straightforward application of MEP with the constraints in Equation (23) and the entropy in Equation (15) [with the constraints in Equation (24) and the entropy in Equation (17) respectively] gives the answers $\hat{\psi }$ in Equation (14) [$\hat{\phi }$ in Equation (16)] to the above questions ($Q_{1})$ and ($Q_{2})$, i.e.,

$$\hat{\psi }\left(\epsilon \right)=\frac{e^{-\beta \epsilon }}{Z(\beta )}=P\left(i\in I_{\epsilon }\right),\;\;\hat{\phi }\left(n\right)=\frac{e^{-\lambda n}}{Z(\lambda )}=P\left(\alpha \in S_{n}\right).$$

Basically, we are solving twice Boltzmann problem of statistical mechanics. Now, if one is interested in the joint probability

(25) $$Q\left(n,\epsilon \right)=P\left(\alpha \in S_{n},i\in I_{\epsilon }\right)$$

we proceed as before, introducing the vector $c=(C_{1,1},\ldots C_{N,M})$ where $C_{n,\epsilon }$ counts the number of times the object (α,i) is assigned to the bin $S_{n}\times I_{\epsilon }$. Hence

(26) $$W\left(c\right)=\frac{1}{{\left(NM\right)}^{N_{0}S}}\frac{(N_{0}S)!}{C_{1,1}!\ldots C_{N,M}!}$$

with

(27) $$\ln W\left(c\right)=-N_{0}S\ln N_{0}S-\sum_{n,\epsilon }C_{n,\epsilon }\ln C_{n,\epsilon }$$

to be maximized under the *decoupled* constraints ($\sum_{\epsilon }C_{n,\epsilon }=S_{n},\;\sum_{n}C_{n,\epsilon }=n_{\epsilon }$)

(28) $$\sum_{n,\epsilon }C_{n,\epsilon }=N_{0}S,\;\sum_{n,\epsilon }nC_{n,\epsilon }=N_{0},\;\sum_{n,\epsilon }\epsilon C_{n,\epsilon }=E_{0}.$$

The related entropy is, setting $C_{n,\epsilon }=Q\left(n,\epsilon \right)N_{0}S$

(29) $$H_{N\times M}\left(Q\right)=-\sum_{n,\epsilon }Q\left(n,\epsilon \right)\ln Q\left(n,\epsilon \right)$$

and the maximum entropy distribution with Constraint (28) can be shown to be

(30) $$Q\left(n,\epsilon \right)=\phi \left(n\right)\psi \left(\epsilon \right)=\frac{e^{-\lambda n}}{Z(\lambda )}\frac{e^{-\beta \epsilon }}{Z(\beta )}.$$

We claim that Equation (30) is the correct solution of the MEP problem dealt with in [5,13] in the sense that the choice of the entropy function adopted is supported by the Boltzmann argument. We do not claim that this solution has a better fit with empirically derived patterns with respect to others but only that is derived in a consistent way. In this solution the two random variables ν and *m* are uncorrelated, therefore the energy and species constraints can be dealt with *separately* giving Equations (14) and (16) or *at the same time*. This is a well known property of MEP: if the constraints concern only the marginals of a joint probability, then the MEP solution is the product of two unidimensional distributions.

## 5. Analysis of the Application of MEP in M.E.T.E.

The starting point of M.E.T.E. theory is the following probability distribution in Equation (31) on N×M introduced in [5], formula (4c). Quoting [5], page 2702 above formula (1a) : “R(n,ϵ) is the probability that if a species is picked at random form the species list, then it has abundance *n*, and if an individual is picked at random from *that species* with abundance *n*, then its metabolic rate is ϵ” (is between [ϵ,ϵ+dϵ] in the original continuum energy formulation, italics is ours)

(31) R(n,ϵ)=ϕ(n)Θ(ϵ|n).

This a delicate point: we think that using “that species” makes the above definition logically inconsistent because if there are more than one species with abundance *n* it is impossible to know which species is being picked and so the probability of picking an individual with a given metabolic rate is not uniquely defined. One can readily convince oneself of this by considering a toy model of the systems along the lines of example in Box 7.1 in [13], page. 144, but with at least two species having equal abundance and containing individuals with the same metabolic rate. The only way for us to be logically consistent is to substitute “that species” with “a species”. This is equivalent to picking an individual of given metabolic rate from the multispecies $I_{n}$. In this paper we have thus intended the definition of R(n,ϵ) as amended in this way.

Note however that in the sequel of [5], below formula (4c), it is (correctly) written “a species”. Note also that the same ambiguity is present in the book [13], Chapter 7, pages 142 and 143. In our framework, we thus rephrase the definition as

(32) $$R\left(n,\epsilon \right)=\phi \left(n\right)P\left(i\in I_{\epsilon }|i\in I_{n}\right)$$

and using the definition of conditional probability P(A,B)=P(A)P(B|A) and (4), (22) also as

(33) $$R\left(n,\epsilon \right)=\phi \left(n\right)\frac{P(i\in I_{\epsilon },i\in I_{n})}{P(i\in I_{n})}=\phi \left(n\right)\frac{P(n,\epsilon )}{\phi_{I}\left(n\right)}=\frac{N_{0}}{Sn}P\left(n,\epsilon \right)$$

Therefore, the probability distributions P(n,ϵ) and R(n,ϵ) are related by the invertible transformation *g* (we use the same symbol for the sake of simplicity)

$$P\left(n,\epsilon \right)=g_{n}\left(R\left(n,\epsilon \right)\right)=\frac{nS}{N_{0}}R\left(n,\epsilon \right).$$

Moreover, the information $N_{0},S,E_{0}$ sets the following constraints on R(n,ϵ), see (1a)–(1c) in [5]

(34) $$\sum_{n,\epsilon }nR\left(n,\epsilon \right)=\frac{N_{0}}{S}$$

(35) $$\sum_{n,\epsilon }\epsilon nR\left(n,\epsilon \right)=\frac{E_{0}}{S}$$

(36) $$\sum_{n,\epsilon }R\left(n,\epsilon \right)=1$$

hence also R(n,ϵ) is properly normalized. In [5], the MEP procedure is applied as follows: the probability distribution R(n,ϵ) is the one that maximize the Shannon entropy

(37) $$H\left(R\right)=-\sum_{n,\epsilon }R\left(n,\epsilon \right)\ln R\left(n,\epsilon \right)$$

on the set defined by Constraints (34)–(36) above. It is therefore the least informative probability distribution on the basis of the sole knowledge of the macroscopic ratios $E_{0}/S$, $N_{0}/S$. Note that, as observed in [5], if the previous ratios are known, $E_{0}/N_{0}$ is also known.

Standard application of the MEP gives the probability distribution depending on the unknown multipliers λ and β (see [5], formula (6))

(38) $$R\left(n,\epsilon \right)=\frac{e^{-\lambda n-\beta \epsilon n}}{Z(\lambda ,\beta )}.$$

The Lagrange multipliers λ and β have to be determined by inserting Equation (38) into the constraints in Equations (34) and (35) but an analytical solution of the resulting equations does not exist. Adopting some approximations in [5] we are lead to the following explicit formulae for the marginal (the SAD distribution)

(39) $$\Phi \left(n\right)=\sum_{\epsilon }R\left(n,\epsilon \right)\approx \frac{1}{\ln\lambda^{-1}}\frac{e^{-\lambda n}}{n}$$

which is the celebrated Fisher log-series ([20]) and to

(40) $$\Psi \left(\epsilon \right)=\frac{S}{N_{0}}\sum_{n}R\left(n,\epsilon \right)\approx \lambda \beta \frac{e^{-\lambda -\beta \epsilon }}{{\left(1-e^{-\lambda -\beta \epsilon }\right)}^{2}}$$

for the energy marginal. Moreover, the conditional probability is

(41) $$\Theta \left(\epsilon |n\right)\approx \beta ne^{-\beta n\epsilon }.$$

What is striking in the above result, is that it prescribes a non zero correlation (Equation (41)) between the distribution of energy among individuals and the distribution of abundance among species, even if in the initial information nothing seems to hint at a possible correlation. This is for us the signal of a flaw in the above application of MEP.

### The Correct Form of Entropy Function of M.E.T.E. Problem

Our aim now is to derive the form of the entropy function for the MEP problem in the R(n,ϵ) variable with constraints on energy and species in Equations (34)–(36) using the Boltzmann argument. Since R(n,ϵ) is not a joint probability distribution, we derive the entropy function for the distribution $P\left(n,\epsilon \right)=P\left(i\in I_{n},i\in I_{\epsilon }\right)$ in (22) which is related to R(n,ϵ) by the change of variable (33) and use the Proposition 1. The *g*-related constraints of Equations (34)–(36) for P(n,ϵ) are

$$\sum_{n,\epsilon }P\left(n,\epsilon \right)=1,\;\sum_{n,\epsilon }\frac{1}{n}P\left(n,\epsilon \right)=\frac{S}{N_{0}},\;\sum_{n,\epsilon }\epsilon P\left(n,\epsilon \right)=\frac{E_{0}}{N_{0}}.$$

Note that using P(n,ϵ) the constraints are only on the marginals of P(n,ϵ) hence unlike for R(n,ϵ) the MEP solution is the product of the marginals, see (48) below. Given the occupation vector $i=(i_{1},\ldots ,i_{N})$ of the $N_{0}$ individuals in the *N* multispecies, for every *n* we have to distribute $i_{n}$ individuals among the *M* energy groups $I_{\epsilon }$ and each arrangement $i_{n}=\left(i_{n,1},\ldots ,i_{n,M}\right)$ with $i_{n}=\sum_{\epsilon }i_{n,\epsilon }$ has probability

(42) $$W_{n}\left(i_{n}\right)=\frac{1}{M^{i_{n}}}\frac{i_{n}!}{i_{n,1}!\ldots i_{n,M}!}$$

Therefore, taking into account that we have $i_{n}/n$ independent tosses in the bin $I_{n}$, we have to use the multiplicity factor W(i) in Equation (19)

(43) $$W=W\left(i\right)\prod_{n=1}^{N}W_{n}\left(i_{n}\right)$$

hence

(44) $$\ln W=\ln W\left(i\right)+\ln\prod_{n=1}^{N}W_{n}\left(i_{n}\right).$$

The maximum of lnW is reached when both terms in the right hand side are maximized, but the first term lnW(i) is independent of the arrangement in the energy levels. Therefore we can maximize the second term in the right hand side of Equation (44) for fixed values of i and then maximize the first term with respect to i. Now

(45) $$\ln\prod_{n=1}^{N}W_{n}\left(i_{n}\right)=\sum_{n}\ln W_{n}\left(i_{n}\right)=-\sum_{n,\epsilon }i_{n,\epsilon }\ln i_{n,\epsilon }+f\left(i\right)$$

is to be maximized under the N+1 constraints (note that the constraint $\sum_{n,\epsilon }i_{n,\epsilon }=N_{0}$ is already enforced since $\sum_{n}i_{n}=N_{0}$)

(46) $$\sum_{n,\epsilon }\epsilon i_{n,\epsilon }=E_{0},\;\mathrm{and}\;\sum_{\epsilon }i_{n,\epsilon }=i_{n},\;n=1,\ldots ,N.$$

The unconstrained extremum problem for the Lagrange function

(47) $$G=-\sum_{n,\epsilon }i_{n,\epsilon }\ln i_{n,\epsilon }-\beta \left(\sum_{n,\epsilon }\epsilon i_{n,\epsilon }-E_{0}\right)-\sum_{n}\mu_{n}\left(\sum_{\epsilon }i_{n,\epsilon }-i_{n}\right)$$

gives the solution

$$i_{n,\epsilon }=i_{n}\frac{e^{-\beta \epsilon }}{\sum_{n}e^{-\beta \epsilon }}=i_{n}\frac{e^{-\beta \epsilon }}{Z(\beta )}.$$

Since $i_{n,\epsilon }=P\left(n,\epsilon \right)N_{0}$ and $i_{n}=\phi_{I}\left(n\right)N_{0}$ we have

(48) $$P\left(n,\epsilon \right)=\phi_{I}\left(n\right)\frac{e^{-\beta \epsilon }}{Z(\beta )}$$

and to get the complete solution we have to maximize W(i), which has already been dealt with in the previous section, Equations (19) and (20). Therefore, the MEP procedure for the distribution P(n,ϵ) with the constraints gives the solution

(49) $$P\left(n,\epsilon \right)=\frac{nS}{N_{0}}\frac{e^{-\lambda n}}{Z(\lambda )}\frac{e^{-\beta \epsilon }}{Z(\beta )}.$$

Using the change of variables in Equation (33) and Proposition 1, we find the solution of the MEP problem in [5] with respect to the variables R(n,ϵ)

(50) $$R\left(n,\epsilon \right)=\frac{N_{0}}{Sn}P\left(n,\epsilon \right)=\frac{e^{-\lambda n}}{Z(\lambda )}\frac{e^{-\beta \epsilon }}{Z(\beta )}$$

which coincides with Q(n,ϵ) in (30).

## 6. Discussion

A non trivial task in the application of the MEP is the translation in mathematical terms (i.e., as constraints on the sought probability distribution) of the information available on the system. In some cases one may hesitate between mathematically equivalent formalizations. What is not so well known is that the use of Shannon entropy H(p) may not be justified in all cases. We have provided a criterion based on Boltzmann method of the most probable occupation vector to derive the correct form of the entropy function in a given formulation. Note that the problem addressed here is different from the search of a suitable prior distribution for the relative entropy D(p|q). In the second part of the paper we have applied this analysis to critically examine the Maximum Entropy Theory of Ecology (M.E.T.E.). Even if the application of MEP contained in M.E.T.E. seems flawed, the resulting SAD proposed by M.E.T.E. is the Fisher log-series, which is widely accepted in the ecological community and considered as giving a best fit for many ecological communities (although the use of log-series SAD has been questioned recently, see [21]). Therefore, the previsions of species abundance based on M.E.T.E. may well be in good agreement with field data. What seems to be hardly giving a sound base in M.E.T.E. is the claim that the information contained in $N_{0},S,E_{0}$ produces a coupling between the distribution of abundance between species and the distribution of metabolic energy between individuals.

## Appendix A

**Proof** **of** **Proposition** **1.** To seek for the constrained extrema of H(p) over the set f(p)−c=0, $c\in R^{k}$, we introduce the Lagrange function

$$G\left(p,\lambda \right)=H\left(p\right)+\sum_{\alpha =1}^{k}\lambda_{\alpha }\left(f_{\alpha }\left(p\right)-c_{\alpha }\right)$$

$\hat{p}$ is a constrained extremum if and only if for all *i*

$${\left(\nabla_{p}G\left(\hat{p},\lambda \right)\right)}_{i}={\left(\nabla H\left(\hat{p}\right)\right)}_{i}+\sum_{\alpha =1}^{k}\lambda_{\alpha }\frac{\partial f_{\alpha }\left(\hat{p}\right)}{\partial p_{i}}={\left(\nabla H\left(\hat{p}\right)+df^{T}\left(\hat{p}\right)\lambda \right)}_{i}=0.$$

Let $H^{\prime }\left(p^{\prime }\right)=H\left(g\left(p^{\prime }\right)\right)$ and $f^{\prime }\left(p^{\prime }\right)=f\left(g\left(p^{\prime }\right)\right)$ where $p=g(p^{\prime })$ is invertible and

$$G^{\prime }\left(p^{\prime },\mu \right)=H^{\prime }\left(p^{\prime }\right)+\sum_{\alpha =1}^{k}\mu_{\alpha }\left(f_{\alpha }^{\prime }\left(p^{\prime }\right)-c_{\alpha }\right).$$

$p^{\prime }$ is a constrained extremum of $G^{\prime }$ over the set $f^{\prime }\left(p^{\prime }\right)=c$ if and only if for all *i*

$${\left(\nabla_{p^{\prime }}G\left(p^{\prime },\mu \right)\right)}_{i}={\left(\nabla H^{\prime }\left(p^{\prime }\right)\right)}_{i}+\sum_{\alpha =1}^{k}\mu_{\alpha }\frac{\partial f_{\alpha }^{\prime }\left(p^{\prime }\right)}{\partial p_{i}^{\prime }}=0$$

that is

$$\sum_{j}\frac{\partial H(g\left(p^{\prime }\right))}{\partial p_{j}}\frac{\partial g_{j}\left(p^{\prime }\right)}{\partial p_{i}^{\prime }}+\sum_{j,\alpha }\mu_{\alpha }\frac{\partial f_{\alpha }\left(g\left(p^{\prime }\right)\right)}{\partial p_{j}}\frac{\partial g_{j}\left(p^{\prime }\right)}{\partial p_{i}^{\prime }}=0$$

or, in compact notation

$$dg^{T}\left(p^{\prime }\right)\left(\nabla_{p}H\left(g\left(p^{\prime }\right)\right)+df^{T}\left(g\left(p^{\prime }\right)\right)\mu \right)=0$$

Since *g* is invertible, detg≠0, and $\nabla_{p}H\left(g\left(p^{\prime }\right)\right)+df^{T}\left(g\left(p^{\prime }\right)\right)\mu =0$ if and only if $\hat{p}=g\left(p^{\prime }\right)$ is a constrained extremum for H(p) and λ=μ. ☐
